# Spontaneous Coronary Artery Dissection (SCAD) Presenting as Non-ST Elevation Myocardial Infarction (NSTEMI) During Menstruation in a Hypertensive Woman: A Case Report of Menstrual Hormonal Fluctuations as a Potential Trigger

**DOI:** 10.7759/cureus.108711

**Published:** 2026-05-12

**Authors:** Nirajan Khati, Rakesh Kumar Shah, Rikesh Karki, Pratima Shakya, Alexandra Ward

**Affiliations:** 1 Internal Medicine, Jersey City Medical Center, Jersey City, USA; 2 Internal Medicine, Detroit Medical Center/Sinai Grace Hospital, Detroit, USA; 3 Internal Medicine, Cambridge Health Alliance, Harvard Medical School, Boston, USA; 4 Internal Medicine, Jalalabad Ragib-Rabeya Medical College, Sylhet, BGD; 5 Cardiovascular Disease, Jersey City Medical Center, Jersey City, USA

**Keywords:** acute coronary syndrome (acs), coronary angiography, hypertension, menstruation, spontaneous coronary artery dissection (scad)

## Abstract

Spontaneous coronary artery dissection (SCAD) is an uncommon, non-atherosclerotic cause of acute coronary syndrome (ACS) that primarily affects younger women. We describe the case of a 33-year-old woman with hypertension who presented with acute chest pain during menstruation and was diagnosed with SCAD by coronary angiography. Hormonal fluctuations during the menstrual cycle have been postulated to contribute to the pathogenesis of SCAD through transient alterations in vascular wall integrity and arterial stability in susceptible individuals. This report highlights the need for heightened clinical suspicion of SCAD in young women presenting with chest pain, particularly in the presence of hypertension and potential hormonal triggers.

## Introduction

Spontaneous coronary artery dissection (SCAD) is an uncommon but increasingly recognized cause of acute coronary syndrome (ACS), primarily affecting younger women without atherosclerotic risk factors [1,2]. It is characterized by a non-atherosclerotic, non-traumatic separation of the coronary arterial wall, resulting in the formation of an intramural hematoma or intimal disruption that can compromise coronary blood flow [2]. SCAD poses a diagnostic challenge because of its variable clinical presentation and the absence of classic ischemic findings [3]. Early recognition is pivotal, as the management of SCAD differs significantly from that of atherosclerotic ACS. Greater awareness of associated and precipitating factors, including hormonal fluctuations associated with menstruation, may facilitate timely diagnosis and appropriate management [4,5]. This report highlights the importance of maintaining a high index of suspicion for SCAD in younger women presenting with symptoms of ACS, particularly in the presence of hypertension and potential hormonal triggers.

## Case presentation

A 33-year-old African American female with a known history of chronic hypertension, first diagnosed at 22 years of age, presented with acute chest pain occurring both during exertion and at rest. She described the chest pain as a mid-sternal, pressure-like sensation radiating to the left jaw and left upper arm, rated 6/10 in intensity. She took ibuprofen without relief of chest pain. In the hospital, she received nitroglycerin with transient improvement; however, the chest pain subsequently recurred. She denied any preceding viral prodrome, including fever, cough, myalgias, and nasal or throat congestion. 

She reported that she was menstruating at the time of presentation with chest pain. She also described a similar episode of chest pain during her previous menstrual cycle, which had resolved spontaneously, for which she had not sought medical attention. She reported that she took labetalol for hypertension inconsistently and did not routinely monitor her blood pressure at home. Her family history was notable for hypertension in her father, who had died of a cerebrovascular accident at the age of 51 years. There was no known family history of premature coronary artery disease or sudden cardiac death. She had experienced four pregnancies, including one live birth at age 17 via normal vaginal delivery and three elective abortions between the ages of 15 and 16 years. She denied any tobacco or illicit drug use and reported only occasional alcohol consumption.

**Figure 1:**
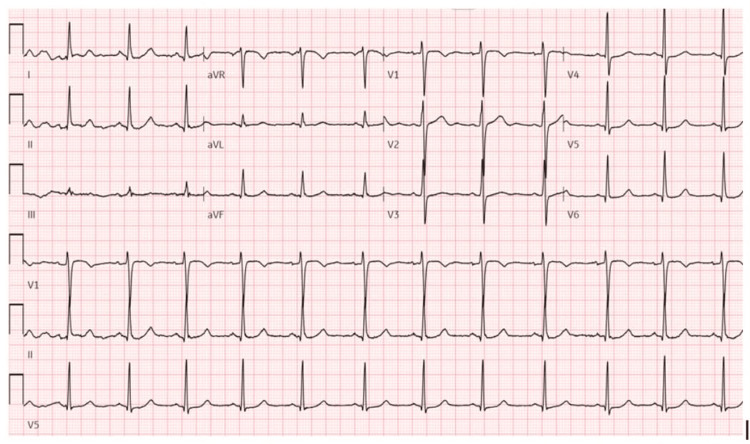
EKG showing normal sinus rhythm with no acute ischemic changes EKG: electrocardiogram

On presentation, her vital signs were stable except for mildly elevated blood pressure (142/84 mmHg), and she was afebrile. Physical examination was unremarkable. Laboratory studies were significant for a high-sensitivity troponin level of 7,000, which subsequently decreased to 6,086 ng/L, and a lipid panel showing an LDL level of 116 mg/dL, as shown in Table 1. The viral respiratory panel was unremarkable. ECG showed no acute ST-segment or T-wave changes, as shown in Figure 1. Chest X-ray was unremarkable. A bedside echocardiogram showed a preserved left ventricular ejection fraction without regional wall motion abnormalities. A formal transthoracic echocardiography (TTE) confirmed these findings, showing a normal ejection fraction, normal diastolic function, and no valvular or wall motion abnormalities. Given her presentation, she was diagnosed with a non-ST elevation myocardial infarction (NSTEMI). She was started on aspirin 75 mg daily, atorvastatin 80 mg daily, and enoxaparin 1 mg/kg twice daily.

**Table 1:** Lab results of the patient BNP: B-type natriuretic peptide; LDL: low-density lipoprotein; HbA1c: glycated hemoglobin; TSH: thyroid-stimulating hormone

Parameters	Results	Reference range
Sodium	141 mmol/L	135 - 145 mmol/L
Potassium	4.2 mmol/L	3.5 - 5.1 mmol/L
Magnesium	2.1 mg/dl	1.6 - 2.6 mg/dl
Phosphorus	3.6 mg/dl	2.4 - 5.1 mg/dl
High-sensitivity troponin I	7,000 (reduced to 6,086) ng/L	3 - 34 ng/L
BNP	84 pg/ml	2 - 100 pg/ml
White blood cell count	7.2 x 10^3^/uL	4.5 - 11 x 10^3^/uL
Total cholesterol	180 mg/dl	25 - 200 mg/dl
Triglycerides	120 mg/dl	10 - 150 mg/dl
LDL	116 mg/dl	5 - 100 mg/dl
HbA1c	5.5%	3.8 - 5.7%
TSH	2.5 mIU/L	0.55 - 4.78 mIU/L

**Figure 2:**
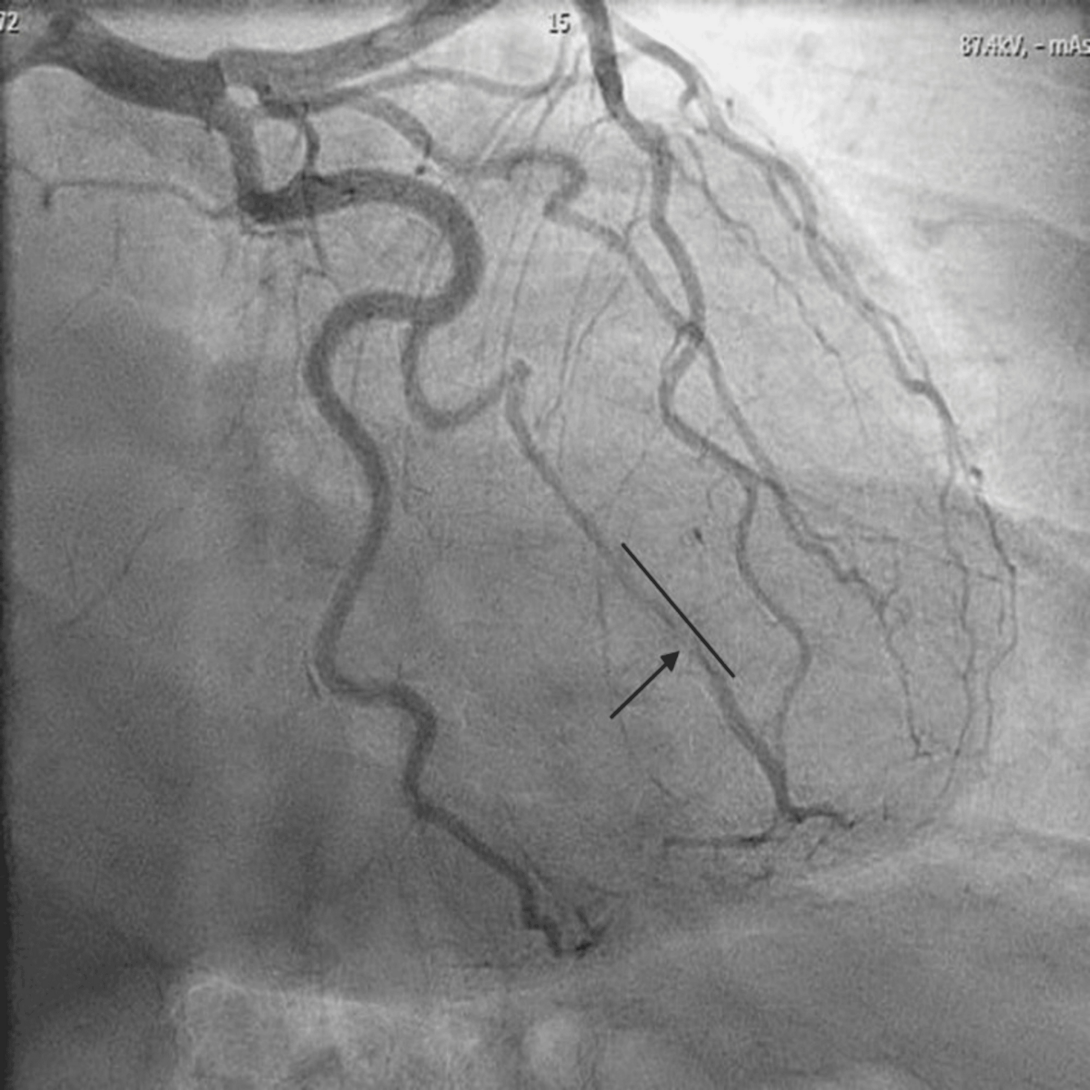
Coronary angiogram demonstrating type 2 SCAD involving the OM2 of the LCx SCAD: spontaneous coronary artery dissection; OM2: second obtuse marginal branch; LCx: left circumflex artery

Cardiology was consulted, and the patient underwent coronary angiography, which revealed type 2 SCAD in the second obtuse marginal branch (OM2) with TIMI 3 flow, as shown in Figure 2. Given the absence of high-risk anatomy and preserved flow, a conservative medical management approach was pursued. To evaluate for fibromuscular dysplasia (FMD), a known condition associated with SCAD, she underwent CT imaging of the head, chest, abdomen, and pelvis, all of which were unremarkable. Her antihypertensive regimen was optimized with labetalol 300 mg twice daily and losartan 50 mg daily.

After three days of inpatient monitoring, the patient was discharged in stable condition with a comprehensive management plan that included aspirin 81 mg daily, atorvastatin 80 mg daily, labetalol 300 mg twice daily, losartan 50 mg daily, and sublingual nitroglycerin as needed for anginal symptoms. She was counseled on the nature of SCAD, its association with hypertension, as well as hormonal and vascular factors, and the importance of medication adherence and lifestyle modification. She was advised to maintain close outpatient follow-up with cardiology for ongoing care and monitoring.

## Discussion

SCAD is a non-atherosclerotic, non-traumatic cause of ACS. It results from the separation of the coronary arterial wall, creating a false lumen which compresses the true lumen, thereby impairing myocardial perfusion [1]. SCAD is recognized as an important cause of myocardial infarction with non-obstructive coronary arteries (MINOCA). Alternative etiologies should be considered in the differential diagnosis of MINOCA, including coronary vasospasm, coronary microvascular dysfunction, coronary thromboembolism, plaque disruption with spontaneous recanalization, myocarditis, and stress-induced cardiomyopathy (Takotsubo syndrome) [6]. 

In our case, these alternative etiologies were considered less likely based on the absence of infectious symptoms or systemic illness, arguing against myocarditis; the lack of characteristic apical ballooning or regional wall motion abnormalities with preserved left ventricular systolic function, arguing against Takotsubo syndrome; the absence of hemodynamic instability or arrhythmias; and definitive angiographic evidence of type 2 SCAD involving the OM2 branch, supporting SCAD as the primary etiology of NSTEMI [2,4]. Recent registry data suggest that SCAD may account for approximately 1-4% of ACS cases overall and up to 35% of ACS cases in women ≤50 years of age [2]. However, it remains underdiagnosed due to its often subtle or atypical clinical presentation [3]. Our patient was a 33-year-old woman with a history of hypertension who presented with acute-onset chest pain typical of myocardial ischemia, described as a midsternal, pressure-like sensation radiating to the left jaw and left arm. The timing of her symptoms during menstruation raises an important consideration regarding hormonal influences as potential triggers for SCAD [4,5].

The pathogenesis of SCAD is not fully understood. It is believed to involve an intimal tear or a spontaneous intramural hematoma leading to compression of the arterial lumen without the presence of atherosclerotic plaque rupture [6]. Several factors have been associated with an increased risk of SCAD, including female sex and hypertension, along with hormonal influences such as those seen during the peripartum and postpartum periods, menstruation, and oral contraceptive use. Additional predisposing conditions include FMD, connective tissue disorders, and exposure to significant emotional or physical stressors [7,8]. In our case, a history of hypertension was notable, as elevated shear stress may have been implicated as a potential trigger in SCAD pathogenesis [9]. However, hypertension is less frequently associated with SCAD than with typical ACS [10]. Additionally, a possible hormonal contribution is supported by the observed association with menstruation. Hormonal variation, particularly the reduction in progesterone levels during the menstrual phase, may adversely affect vascular wall stability and predispose susceptible individuals to arterial dissection [11].

Initial evaluation typically includes ECG, cardiac biomarkers such as troponin, and TTE. In this case, the ECG did not demonstrate ischemic changes. Although elevated troponin levels indicated myocardial injury, the absence of regional wall motion abnormalities on TTE may reflect a limited or subendocardial extent of infarction [12]. Coronary angiography, considered the diagnostic gold standard, confirmed the presence of SCAD [13]. A conservative management approach was chosen in accordance with current recommendations, as percutaneous coronary intervention (PCI) in SCAD is associated with increased technical difficulty and a higher risk of complications, including extension of the dissection due to the fragility of the arterial wall [14]. Given our patient's hemodynamic stability and absence of high-risk characteristics such as left main coronary involvement or persistent ischemia, a conservative medical approach was deemed appropriate [1,13]. Hemodynamic instability, active ischemia, and high-risk features warrant consideration for revascularization with PCI or coronary artery bypass grafting (CABG) [2].

The medical management of SCAD primarily involves antiplatelet therapy. Dual antiplatelet therapy (DAPT) is generally limited to patients who have received coronary stents. Statin therapy is not routinely indicated unless there is an additional indication, such as established atherosclerotic cardiovascular disease or dyslipidemia. Beta-blockers are commonly recommended based on observational evidence suggesting a potential reduction in SCAD recurrence [13]. A comprehensive CT (pan-CT) evaluation may be performed to screen for associated systemic arteriopathies, including fibromuscular dysplasia, which is present in up to 80% of SCAD patients and may warrant surveillance of extracoronary vessels [7,9]. CT of the chest, abdomen, and pelvis revealed no abnormalities, and CT of the head was also unremarkable. Although no evidence of FMD or connective tissue disorders was found, ongoing clinical monitoring and possibly outpatient genetic counseling may be considered given the family history of her father's death from a cerebrovascular accident at the relatively young age of 51 years [5].

The prognosis of SCAD is generally favorable when diagnosed early and managed appropriately [1,6]. However, recurrence remains a concern, occurring in approximately 10-30% of patients [2,14]. In addition, some patients may experience persistent chest pain as well as psychological effects such as anxiety and depression following the acute event [9,15]. Education and counseling are crucial for patient-centered care, and cardiac rehabilitation has been shown to improve both physical recovery and psychological outcomes in patients with SCAD [15]. Our patient showed a good response to medical therapy, with complete resolution of chest pain and no recurrence of ischemic symptoms during her hospital stay.

## Conclusions

The report underscores the importance of considering SCAD in younger women with a history of hypertension who present with symptoms of ACS, particularly when these symptoms coincide with hormonal fluctuations such as those occurring during menstruation. A high index of suspicion, along with prompt coronary angiography, is essential for diagnosis. Medical management remains the cornerstone of treatment in stable patients.
